# BREX system of *Escherichia coli* distinguishes self from non-self by methylation of a specific DNA site

**DOI:** 10.1093/nar/gky1125

**Published:** 2018-11-12

**Authors:** Julia Gordeeva, Natalya Morozova, Nicolas Sierro, Artem Isaev, Tomas Sinkunas, Ksenia Tsvetkova, Mikhail Matlashov, Lidija Truncaitė, Richard D Morgan, Nikolai V Ivanov, Virgis Siksnys, Lanying Zeng, Konstantin Severinov

**Affiliations:** 1 Skolkovo Institute of Science and Technology, Moscow 143028, Russia; 2 Peter the Great St Petersburg State Polytechnic University, St Petersburg 195251, Russia; 3 Philip Morris International R&D, Philip Morris Products S.A., Neuchâtel 2000, Switzerland; 4 Institute of Biotechnology, Vilnius University, Saulėtekio Avenue 7, Vilnius 10257, Lithuania; 5 Institute of Biochemistry, Vilnius University, Saulėtekio Avenue 7, Vilnius 10257, Lithuania; 6 New England Biolabs, 240 County Road, Ipswich, MA 01938, USA; 7 Department of Biochemistry and Biophysics, Center for Phage Technology, Texas A&M University, College Station, TX 77843, USA; 8 Waksman Institute of Microbiology, Piscataway, NJ 08854, USA

## Abstract

Prokaryotes evolved numerous systems that defend against predation by bacteriophages. In addition to well-known restriction-modification and CRISPR-Cas immunity systems, many poorly characterized systems exist. One class of such systems, named BREX, consists of a putative phosphatase, a methyltransferase and four other proteins. A *Bacillus cereus* BREX system provides resistance to several unrelated phages and leads to modification of specific motif in host DNA. Here, we study the action of BREX system from a natural *Escherichia coli* isolate. We show that while it makes cells resistant to phage λ infection, induction of λ prophage from cells carrying BREX leads to production of viruses that overcome the defense. The induced phage DNA contains a methylated adenine residue in a specific motif. The same modification is found in the genome of BREX-carrying cells. The results establish, for the first time, that immunity to BREX system defense is provided by an epigenetic modification.

## INTRODUCTION

Bacteriophages outnumber their hosts in nature and can have major effects on bacterial populations and communities. For example, recent analysis demonstrates massive phage-driven sweeps in oceanic bacterial communities (1,2). In response to viral predation cellular defense mechanisms have evolved to prevent annihilation. They include, among others, innate immunity conferred by restriction-modification systems (3,4), adaptive immunity mediated by CRISPR-Cas systems (5–7) and an assortment of poorly studied abortive infection mechanisms that limit phage propagation through the population while killing infected cells (8–10). Considering the diversity of phages and the fact that defense islands constitute ∼10% of bacterial genomes (11), there is little doubt that multiple host resistance systems remain to be discovered and recent work supports this view (12). In addition, a number of defense systems were reported in the sixties and seventies of the last century but were largely forgotten. One such system is Pgl (phage growth limitation) of *Streptomyces coelicolor* that affects the growth of phage φC31 (13–15). While there is no difference in burst sizes and lysis times of φC31-infected Pgl+ and Pgl− cells, in continuously infected cultures the titer of the phage is orders of magnitude lower in Pgl+ cultures compared to Pgl− cultures. It was proposed that phages released after the first round of Pgl+ cells infection become modified and lose the ability to infect Pgl+ (but not Pgl−) cells.

The Pgl system consists of four genes *pglWXYZ* (14,16,17). Bioinformatics analysis suggested that PglW may be a kinase, PglX—a SAM-dependent methyltransferase, PglY—an adenosinetriphosphatase (ATPase) and PglZ—an alkaline phosphatase. For PglW, PglX and PglY these predictions were confirmed *in vitro* (15). The current model of the Pgl system function posits that PglX and PglZ act as a toxin-antitoxin pair and that the release of toxic PglX in φC31-infected cells is controlled by PglZ and leads to formation of modifying/restricting complexes (15).

Sequence analysis of prokaryotic defense islands revealed overrepresentation of genes encoding PglZ homologs (16) and a separate BREX (bacteriophage exclusion) name for clusters containing *pglZ*-like genes was recently proposed (17). Introduction of six-gene BREX system from *Bacillus cereus* into a host without its own *brx* genes caused increased levels of resistance to diverse phages. While no effects on phage adsorption were revealed, phage replication was blocked. In addition, host DNA in BREX+ cells was modified by methylation of the fifth adenine residue in a TAGGAG motif. Counterintuitively, the motif was unmethylated in the genome of phage recovered from BREX+ infections.

Here, we explore BREX system from a natural isolate of *Escherichia coli*. We show that it offers protection against phage infection and that the genomes of phages induced from BREX+ cells become modified at an asymmetric site that is also modified in the BREX+ host genome. Critically, modified phages become resistant to BREX defense. We also show that glycosylation of phage genome abolishes BREX defense. While the actual mechanism of BREX protection remains unknown, our results prove that the *E. coli* BREX, and by extension, other systems of this class, distinguish self from non-self by epigenetic modification of DNA similar to R-M systems.

## MATERIALS AND METHODS

### Disruption of the BREX^Ec^ cluster and *pglX* deletion in *E. coli* HS

The BREX cluster was deleted using a Red recombinase-based procedure (18). Briefly, the chloramphenicol resistant gene (*cat*) was amplified from pKD3 plasmid using BREX_CM_F and BREX_CM_R primers (all primers are listed in Supplementary Table S1). Polymerase chain reaction (PCR) product was gel-purified and suspended in elution buffer. Electrocompetent *E. coli* HS cells (19,20) carrying an arabinose-inducible Red λ recombinase plasmid pKD46 (AmpR) were transformed with the purified PCR fragment. Shocked cells were combined with 1 ml LB media and incubated 3 h at 37°C with shaking. Cells were plated on media with chloramphenicol. Colonies with disrupted BREX cluster were verified by PCR using CM_check_F/CM_check_R and BREX_check_F/BREX_check_R primer pairs.

The *pglX* gene was deleted in *E. coli* HS using a two-plasmid Cas9-based system – pTarget-ΔpglX and pCas (Addgene no. 62225). pTarget-ΔpglX plasmid was constructed by modifying pTargetF plasmid (Addgene no. 62226): (i) the DNA fragment containing *pglX*-targeting spacer was amplified using TS578/TS579 primers and pTargetF as template then inserted into the vector via BcuI/XhoI sites; (ii) DNA fragment, which served as recombination template for *pglX* deletion, was amplified with TS580/TS581 and TS582/TS583 primers and inserted into the SalI site. Procedure of the *pglX* gene deletion was performed as described elsewhere (21).

### Cloning of BREX^Ec^ genes

A 14 kbp fragment of *E. coli* HS genome (positions 340 559–354 275, NC_009800.1) containing the entire set of six BREX genes was amplified from using Brx_pro_SacI_F and BrxL_SphI_R primers. The vector backbone was constructed by amplification of the low copy number pBBR1 origin and kanamycin resistance gene from pBTB-2 (22) plasmid using pBTB_SphI_F and pBTB_SacI_R primers. After treating with appropriate restriction endonucleases the vector and BREX fragments were ligated and transformed into *E. coli* DH5α.

Another version of pBREXAL vector was constructed by inserting BREX genes into pACYC184 vector via Eco32I/EheI sites. In this case, DNA fragment containing BREX genes was amplified from *E. coli* HS genome (positions 340 696–354 491, NC_009800.1) using TS551/TS552 primers. This pBREXAL plasmid was used in plaque assays using phages T4, T4147 (unglycosylated 5-hmC-containing T4 mutant was kindly provided by Dr Lindsay W. Black (23)), vB_EcoM_VR5 (VR5), vB_EcoM_VR7 (VR7) (24) and vB_EcoM_VpaE1 (VpaE1) (25) phages.

For experiments with fluorescent phage lambda (Kan^R^) induction, the BREX^Ec^ system was cloned on pTG plasmid. This vector was constructed by ligation of a fragment of the p15A origin and chloramphenicol resistance gene amplified from pACYC184 (using primers pACYC184_F and pACYC184_R) and a DNA fragment containing the arabinose promoter amplified from the pBAD30 plasmid (using primers pBAD30_F and pBAD30_R). The BREX^Ec^ fragment used to construct pBREXAL was ligated with pTG fragment amplified using pTG-BREX_F and pTG-BREX_R primers.

To create a two-plasmid arabinose-inducible BREX^Ec^ system, *brxA, brxB* and *brxC* were amplified together using EcoRI_SD8_BrxA_F and BrxC_EcoRI_R primers and cloned on pBTB-2 resulting in the pBREX1 plasmid. A fragment containing *brxX, brxZ* and *brxL* was cloned on the pBAD-HisB (resulting in plasmid pBREX2) in two stages using BrxZ_NcoI_F/BrxL_XhoI_R and NcoI_BrxX_F/BrxZ_NcoI_R primer pairs.

Deletions of *brxA* and *brxC* were created using outside PCR amplification of pBREX1 by delA_BglII_F/delA_BglII_R and delC_BglII_F/delC_BglII_R primer pairs, respectively. Internal fragments of *brxX* and *brxL* genes were deleted from pBREX2 by, correspondingly, SpeI/SdaI and BglII restriction endonuclease digestion and religation. Deletions of *brxB* and *brxZ* were constructed by overlap extension PCR (26) with delB_F1/delB_R1 and delB_F2/ delB_R2, and delZ_F1/delZ_R1 and delZ_F2/ delZ_R2 primers.

To construct individual *brx* genes expression plasmids, each gene was amplified and cloned on the pBAD-HisB plasmid under the control of arabinose promoter using the Nhe-Brx*N*-dir + XhoStSac-Brx*N-*rev primers, where *‘N’* denotes sequences specific for the beginning and the end of each of the *brx* genes open reading frames (see Supplementary Table S1).

All plasmids were verified by DNA sequencing.

### Plasmid transformation assays

Competent *E. coli* BW25113 (*F^−^, DE(araD-araB)567, lacZ4787(del)::rrnB-3, LAM^−^, rph-1, DE(rhaD-rhaB)568, hsdR514*) cells were prepared using the standard protocol (27) and transformed with 25 ng of pBTB-2 or pBREXAL plasmids. After 1.5 h of incubation at 37°C, the mixture was serially diluted and plated on LB (to measure the number of living cells) and LB + kanamycin (to measure the amount of transformed cells) media. Plates were incubated at 37°C overnight. For each plasmid, transformation was repeated three times. Transformation efficiency was calculated as a ratio of antibiotic-resistant transformant colonies to the total number of colony forming units formed on LB plates.

### Efficiency of plaquing (EOP) assay

Cell cultures were grown until OD_600_ = 0.6 in LBMM medium (LB supplemented with 10 mM MgSO_4_ and 0.2% maltose) with the addition of 50 μg/ml kanamycin, mixed with soft (0.6%) LBMM agar and poured on the surface of precast LBMM 1.5% agar plates. A total of 10 μl aliquots of phage lysates or their serial (10^−1^–10^−8^) dilutions were deposited in drops on the surface of freshly poured lawns. After 18 h of incubation at 37°C, efficiency of plaquing was determined as a ratio of phage titers on BREX+ to BREX− lawns.

Phages T4 and T4147 were propagated in *E. coli* DH10B BREX− and BREX+ cells at 37°C in LB medium. VR5 and VR7 were propagated in *E. coli* BL21 (DE3) BREX− and BREX+ cells at 30°C, while VpaE1 at 37°C in LB.

### Growth curves of infected cultures

Overnight cultures were diluted 1:100 into LBMM supplemented with appropriate antibiotic and grown at 37°C until OD_600_ = 0.6. Phage λ cI*857 bor::Cm* was added to reach appropriate MOI and growth was monitored using EnSpire Multimode Plate Reader (PerkinElmer). At various times post-infection (0, 80 and 180 min), aliquots from infected cultures were taken to determine phage titer (PFU) and the number of living cells (CFU).

### Adsorption assay

Overnight cultures of BREX+ and BREX− cells were diluted 1:100 in LBMM with kanamycin. Cultures were grown until OD_600_ = 0.6, mixed with phage λ cI*857 bor::Cm* at MOI = 0.002 and placed in a rotary shaker at 37°C. A total of 100 μl culture aliquots were withdrawn at various times post-infection (0, 1, 3, 7, 15, 25 min), cells were pelleted by centrifugation at 10 000 × *g* for 3 min and the titer of unabsorbed phage in the supernatant was determined on BREX− cell lawns. Percentage of unadsorbed phages was calculated assuming the initial titer of phage (in the absence of added cells) as 100%.

### Lysogenization assay

In total, 1 ml of overnight cultures of BREX+ and BREX− cells were diluted 1:100 in LBMM media with kanamycin, cultivated for 4 h at 37°C, mixed with phage λ *cI857 bor::Cm* at MOI of 1 to 5 and placed at 30°C. After 1-h incubation, the bacteria-phage mixtures were serially diluted and plated on LB plates supplemented with kanamycin and kanamycin + chloramphenicol followed by overnight growth at 30°C. Lysogenization frequency was calculated as a ratio of the number of colonies grown on kanamycin + chloramphenicol plates to the number of colonies formed on plates with kanamycin only.

### Fluorescence microscopy

#### Visualization of phage lambda induction

Overnight cultures of LE392(λ_LZ1_) lysogens (28) transformed with pTG or pTG-BREX plasmids were diluted 1:100 and cultivated in LB with chloramphenicol at 30°C. When OD_600_ reached 0.4, cultures were transferred to 42°C for 15 min to trigger phage induction. Culture aliquots (1 ml) were centrifuged for 3 min at 4300 × *g*, cells were diluted in 300 μl LB and 1 μl was placed on an LB + 1.5% agarose slab (∼1 mm thick) resting on a large 24 × 50 mm coverslip (Fisher Scientific). After 1-min drying the slab was covered by a small 18 × 18 mm coverslip (Fisher Scientific).

#### Visualization of injected phage DNA

The procedure is described in detail elsewhere (29). Briefly, overnight cultures of LZ204 (29) transformed with pBTB-2 or pBREXAL plasmids were diluted 1:100 in M9 + 0.4% maltose (M9M) with kanamycin and allowed to grow at 37°C until OD_600_ reached 0.4. 1-ml culture aliquots were centrifuged at 6000 rpm for 3 min and cells were resuspended in 150 μl of cold M9M. For experiments with propidium iodide staining, bacterial culture was mixed with propidium iodide to the final concentration of 20 μM. Fluorescent phage stock (10 μl, 10^7^ PFUs) was mixed with the same volume of cells to reach an MOI of ∼1 and incubated on ice for 30 min. The phage-cells mixture was diluted 1:3 in cold M9M and placed to 35°C for 5 min to trigger phage DNA injection. A 1 μl aliquot of the mixture was placed on 1.5% agarose M9M/kanamycin slab as described above.

Fluorescent phages were prepared based on standard protocols (28). Lysogenic culture of cells carrying the λ*D-eyfp cI*_857_*bor::KanR* prophage and a plasmid expressing the wild-type λ gpD capsid protein (to avoid capsid instability) was transformed with pTG or pTG-BREX plasmids. BREX+ and BREX- phages were produced by heat induction from lysogenic cultures and purified as described previously (28) using ultracentrifugation in CsCl gradient.

Imaging was performed on a Nikon Eclipse Ti inverted epifluorescence microscope (29). Either 8 or 16 stages were used for time-lapse movies. In the first frame of the movie, phage was visualized via z-stacks (±1.2 μm, 0.3 μm each step) with EYFP filter (200 ms explosure). During the movie, the sample was imaged in phase contrast for cells detection (100 ms), EYFP for phage (100 ms) and ECFP for SeqA (30 ms) channels.

### PacBio sequencing

Phages and bacterial genomic DNA were purified using phenol-chloroform extraction (30) and Thermo Scientific Genomic DNA Purification kit, respectively. Extracted DNA was sheared to a mean size of 500 bp using an ultrasonicator (Covaris) and purified with AMPure PB beads (Pacific Biosciences). PacBio sequencing libraries were prepared using the SMRTbell Template Prep kit 1.0 (Pacific Biosciences). Protocols for polymerase binding were generated by the Pacific Biosciences Binding Calculator. Sequencing was performed on the PacBio RS II (Pacific Biosciences). The PacBio SMRT Analysis software was used for reads alignment and modification-motif searches.

### Phage DNA isolation and analysis

Aliquots (100–150 μl) of phage suspensions (10^11^–10^12^ PFU/ml) were subjected to phenol/chloroform extraction and ethanol precipitation. Isolated phage DNA was used for restriction analysis with Eco32I, MboI, EcoRII, SalI and Csp6I restriction endonucleases (Thermo Fisher Scientific) according to supplier’s recommendations. *In vitro* DNA glycosylation tests were performed in the Epi Buffer using T4 phage β-glucosyltransferase (T4 BGT) and UDP-glucose from the EpiJET 5-hmC and 5-mC Analysis Kit (Thermo Fisher Scientific). DNA fragments were separated by electrophoresis in a 0.8% agarose gels stained with ethidium bromide.

### Restriction endonuclease activity assay

BREX+ or BREX- cells were incubated overnight without shaking at 37°C. *Escherichia coli* K12 BW25113 with the EcoRV R-M system components expressed from the pEF42 plasmid (31) was used as a positive control. To prepare crude lysates 1 ml of overnight culture was spun down by centrifugation, resuspended in 1 ml of buffer (40 mM Tris–HCl, pH 7.5,150 mM NaCl, 1 mM ethylenediaminetetraacetic acid, 7 mM β-mercaptoethanol) and disrupted by sonication with a brief (5–10 s) impulse. Reactions were carried out in 20 μl volume with 2 μl of crude cell extracts and 200 ng of phage λ DNA for 30 min at 37°C using the following buffer: 10 mM Tris–HCl pH 7.5, 50 mM NaCl, 10 mM MgCl_2_, 0.1 mg/ml bovine serum albumin with optional addition of ATP to 1 mM.

## RESULTS

### *Escherichia coli* HS BREX system provides defense against phage λ infection

**Figure 1.**
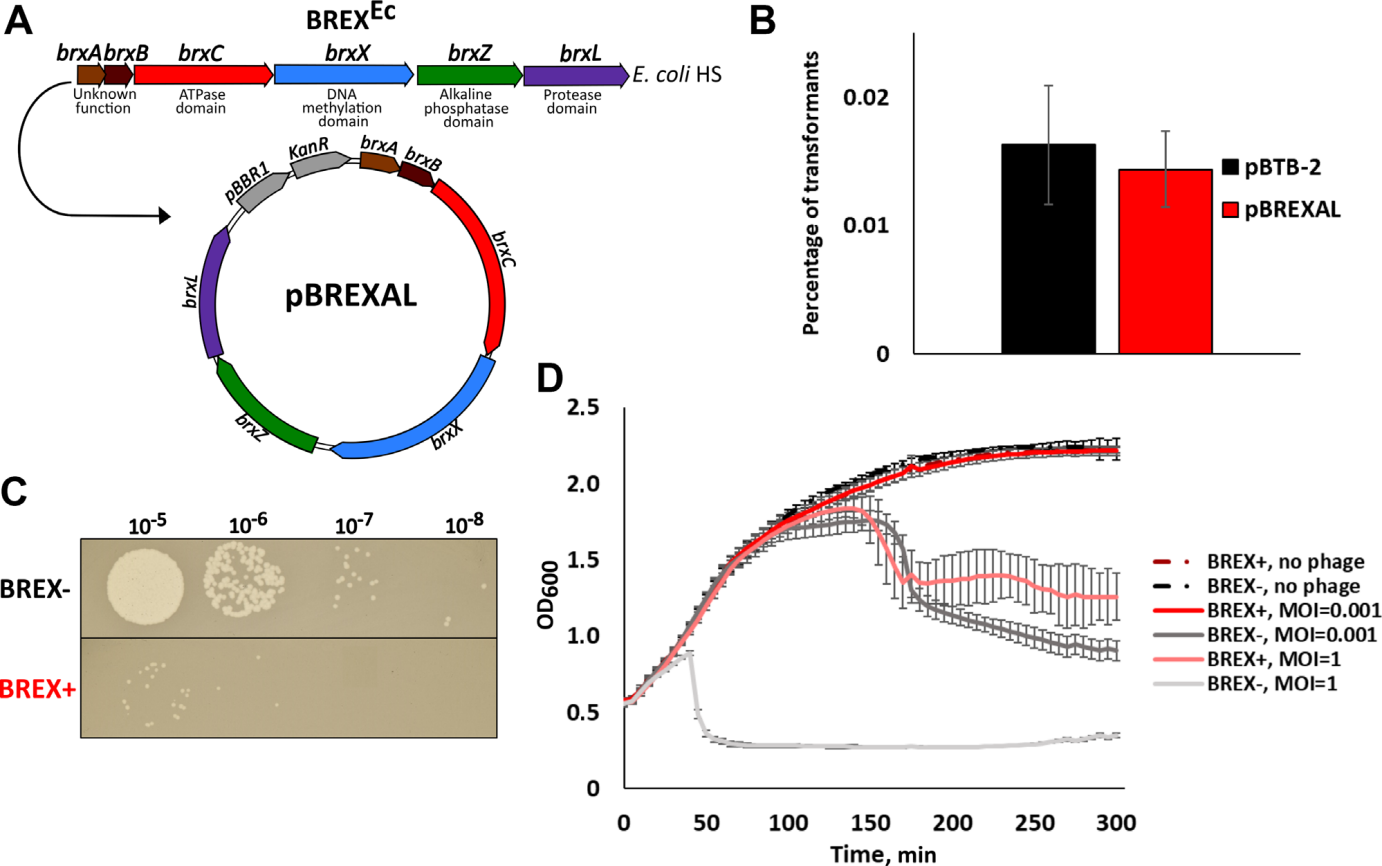
BREX^Ec^ protects cells from phage λ infection. (**A**) The BREX gene cluster from *Escherichia coli* HS is schematically shown; bioinformatically predicted putative functions of *brx* gene products are listed. The entire BREX^Ec^ cluster was cloned into the pBTB-2 vector to yield the pBREXAL plasmid. (**B**) Efficiency of transformation of the empty pBTB-2 vector and the pBREXAL plasmid into laboratory BW25113 *E. coli* to generate, correspondingly, BREX- and BREX+ cells. Mean values from three independent experiments are presented with standard deviations shown. *P*-value is 0.59. (**C**) Lawns formed by BREX+ and BREX- *E. coli* cells were spotted with indicated dilutions of phage λ lysate. Results of overnight growth at 37°C are shown. (**D**) Growth curves of BREX+ and BREX- *E. coli* cultures in the absence of infection, and during infection with λ phage at MOI of 0.001 and 1. Phage was added at *t* = 0. Each growth curve shows mean optical density values and standard deviations obtained from three independent experiments.

Closely related six-gene *brxABCXZL* clusters (Figure 1A) are present in multiple *E. coli* isolates (17). *Escherichia coli* HS, a natural isolate with the *brxABCXZL* cluster, was infected with several phages (λ, T4, T5 and T7). As controls, isogenic strains lacking the entire *brxABCXZL* cluster, or lacking the *brxX* putative methyltransferase gene, were used. All strains were found to be fully resistant to all phages tested. Thus, no conclusions about the contribution of *brx* cluster to phage resistance could be made. The entire *E. coli* HS *brx* cluster was therefore cloned, together with upstream sequences, on a low-copy *E. coli* pBTB-2 plasmid. The resulting plasmid was named pBREXAL (Figure 1A). Both pBTB-2 and pBREXAL plasmids transformed laboratory *E. coli* K12 strain BW25113 with equal efficiency (Figure 1B). Below, we refer to *E. coli* HS cluster as BREX^Ec^; BW25113 cells carrying plasmid-borne BREX^Ec^ are referred to as BREX+; control cells carrying the pBTB-2 vector are referred to as BREX-.

A modified λ *cI*_857_*bor:Cm* phage carrying a *cI*_857_ mutation in the *cI* gene coding for phage repressor, and marked with a chloramphenicol resistance gene that replaced the *bor* gene (encoding phage outer membrane lipoprotein) was obtained by thermal induction from *E. coli* MC4100 lysogen and tested for its ability to form plaques on lawns of BREX+ and BREX- cells or infect liquid cultures. As can be seen from Figure 1C, the efficiency of plaque formation was reduced ∼100-fold in the presence of BREX^Ec^. In the absence of infection, the growth rates of BREX+ and BREX- cells in liquid culture were the same (Figure 1D). In cultures infected with phage λ at low multiplicity of infection (MOI) of 0.001, the BREX- culture collapsed ∼180 min post-infection. In contrast, the BREX+ culture continued to grow at the same rate as the uninfected culture (Figure 1D). At MOI of 1, BREX- cultures lysed 50 min post-infection, while BREX+ cultures continued to grow at the rate of uninfected control for ∼100 min. At later times the growth ceased and optical density slowly declined (Figure 1D). Overall, we conclude that similarly to the BREX system from *B. cereus*, BREX^Ec^ is functional in phage defense.

**Figure 2.**
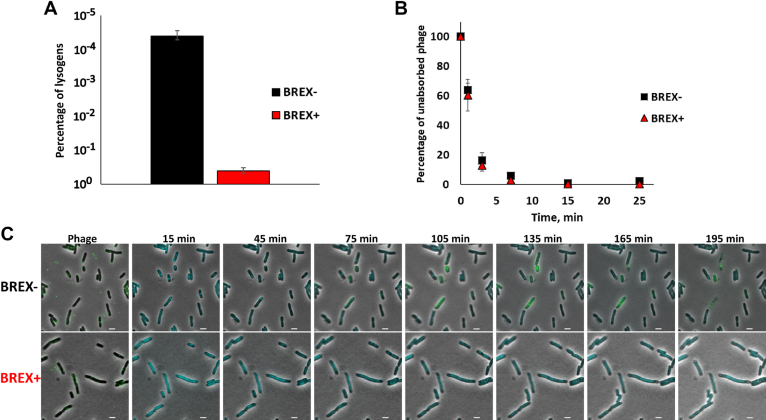
BREX^Ec^ effects on lysogenization, adsorption and DNA injection by bacteriophage λ. (**A**) The bars show the numbers of chloramphenicol-resistant lysogenic colonies formed after high-MOI infection (MOI = 1) of BREX+ and BREX- cells with λ phage marked with a chloramphenicol resistance cassette. Mean values from three independent experiments are presented with standard deviations shown. (**B**) The experiment was conducted as in (A) but at an MOI of 0.01. At indicated times, cells were removed by centrifugation and unadsorbed infectious phage particles in the supernatant were determined on BREX- cells lawns. Mean values from three independent experiments are presented with standard deviations shown. *P*-value is 0.93. (**C**) Live microscopy of BREX- and BREX+ cells infected with phage λ. Images from a time-lapse movie show phage DNA injection. In the first picture, the fluorescent phage appears as a green dot on the cell surface. At 15 min, the SeqA-ECFP foci accumulate as one or two cyan dots, representing the ejected and replicated phage DNA respectively. Scale bar, 2 μm.

The experiments described above tested for the ability of BREX^Ec^ system to affect the lytic pathway of phage λ development that requires phage progeny formation. To determine if BREX^Ec^ also affects lysogenization, BREX+ and BREX- cultures were infected at high MOI and cells were plated on selective media containing chloramphenicol, where only lysogenized cells could form colonies. There was a strong (10^4^-fold) suppression of chloramphenicol-resistant colony formation when BREX+ cultures were infected (Figure 2A). Since lysogenization does not require phage DNA replication (32), the result indicates that defensive action of BREX^Ec^ manifests itself either at the stage of phage adsorption or during injection of phage DNA. Measurements of the dynamics of unadsorbed phages in the course of lysogenization experiment revealed that the adsorption rate was the same in BREX+ and BREX- cultures (Figure 2B). A similar observation was made when phage adsorption to *Bacillus subtilis* cells with and without BREX system was monitored (17).

The process of infection was also monitored by live fluorescence microscopy using phage λ encoding capsid-decoration protein fused to EYFP. The host cells used for infection expressed a fused SeqA::ECFP protein and were *dam−*. SeqA specifically binds to DNA containing methylated or hemimethylated dam sites. As is shown elsewhere (29,33) this property allows one to monitor the process of infection by phage λ containing Dam-methylated DNA by observing fluorescent SeqA binding to injected phage genome, since bound SeqA forms distinct fluorescent foci on injected viral DNA. Immediately upon injection, one fluorescent dot is seen in infected cells. At later stages two dots corresponding to hemimethylated phage genomes are observed (29,33). Under our conditions (MOI = 1–5), phage DNA was injected in ∼53% of 853 BREX- cells analyzed as judged by the appearance of at least one SeqA focus. In some infected cells two SeqA foci appeared, followed by accumulation of yellow fluorescence due to the synthesis of capsid decoration protein::EYFP fusion. Initially diffuse, the EYFP fluorescence subsequently accumulated in speckles likely corresponding to phage capsids and cells lysed shortly thereafter (Figure 2C). BREX- cells where only one SeqA focus was observed survived and likely underwent lysogenic conversion. When BREX+ cells were infected, no ECFP foci or EYFP fluorescence accumulation was observed. The results suggest that phage DNA is either not injected in BREX+ cells or is rapidly degraded once inside the cells. Interestingly, the infected BREX+ cells stopped dividing and ∼18% of 911 cells examined became mildly elongated. Judging by the lack of propidium iodide staining, the membrane of infected BREX+ cells remained intact and no drop in CFU was observed when infected cells were deposited on agar plates, so activation of BREX, if it happens upon infection, does not kill cells.

### BREX^Ec^ has no effect on lysogenic induction

**Figure 3.**
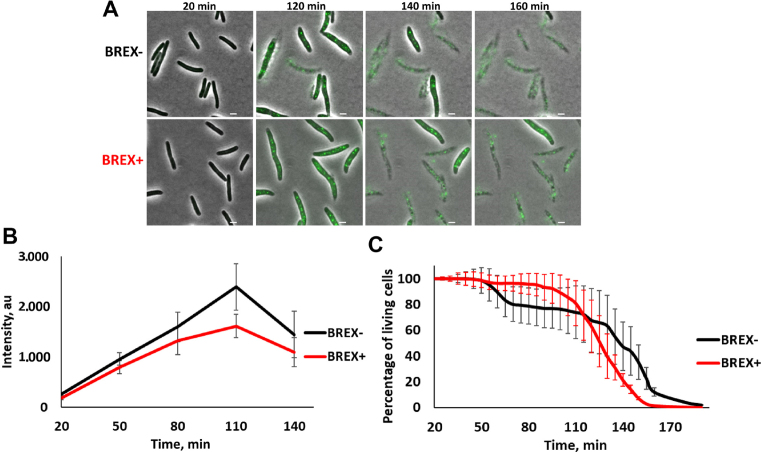
Induction of λ prophage from BREX+ lysogens. (**A**) Images of cells from BREX- and BREX+ lysogenic cultures taken at indicated times after thermal induction. The induced phage encodes capsid decoration protein fused to EYFP. Scale bar, 2 μm. (**B**) Changes in EYFP fluorescence in induced cells are presented at various times post-induction. Mean numbers were calculated from fluorescence intensities obtained with ca. 300 cells. Standard deviations of mean numbers obtained in three independent experiments are shown. *P*-value is 0.46. (**C**) Quantification of a representative kinetic series showing decrease in live cells during microscopic observation of induced lysogenic cultures. Mean values are presented and standard deviations shown. *P*-value is 0.91.

*Escherichia coli* cells lysogenized with λ*D-EYFP cI*_857_*bor::Kan^R^* were transformed with a plasmid with the BREX^Ec^ system or empty vector control. The resulting BREX+ and BREX- lysogenic cultures were shifted to 42°C to induce the prophage, transferred to 30°C and monitored over time using live fluorescent microscopy (Figure 3A). Preliminary experiments showed that BREX protection was as effective at 42°C as it was at 30 and 37°C (Supplementary Figure S2). Shortly after the induction both BREX+ and BREX- cells became fluorescent indicating accumulation of the decoration protein::EYFP fusion. At later times, fluorescent speckles corresponding to assembled phage capsids appeared inside the cells. The intensity of diffuse cytoplasmic fluorescence and the number of dots were nearly the same in BREX+ and BREX- cells (Figure 3B), indicating comparable phage yield. Most cells in both induced cultures lysed ∼3 h post-induction and the kinetics of cell lysis was the same in BREX+ and BREX- cultures (Figure 3C). We therefore conclude that BREX^Ec^ has no effect on lysogenic induction.

**Figure 4.**
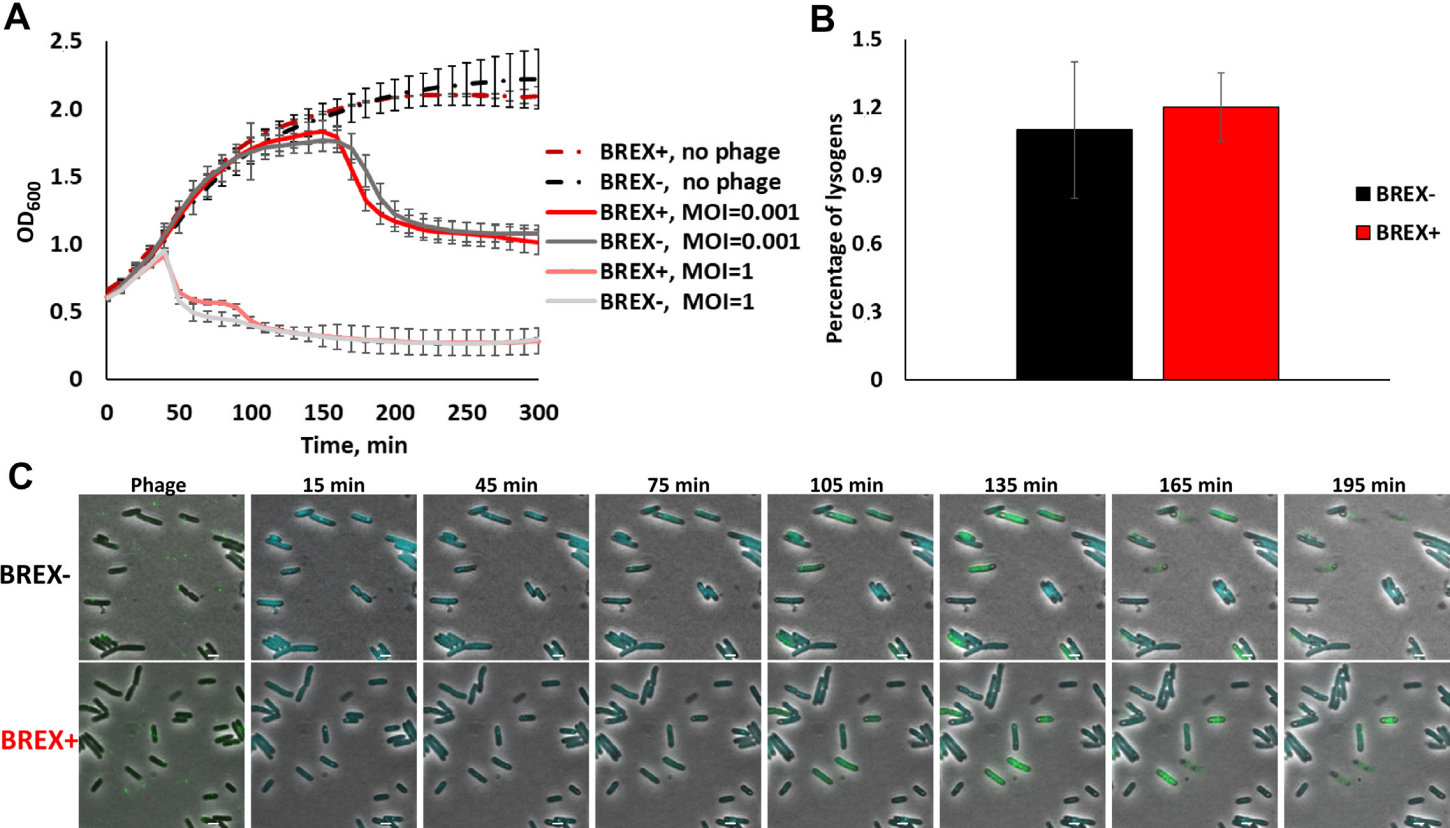
λ phage induced from BREX+ lysogens is not subject to inhibitory action of BREX^Ec^. (**A**) Growth curves of liquid cultures of BREX+ and BREX- cells in the absence of infection, and during infection with λ phage obtained after induction of BREX+ lysogens. Results of infections at MOI 0.001 and 1 are presented. In infected cultures, phage was added at *t* = 0. Mean values from three independent experiments are presented with standard deviations shown. (**B**) The bars show the number of chloramphenicol-resistant lysogenic colonies formed after infection (MOI = 5) of BREX+ and BREX- cells with phage λ phage obtained after induction of BREX+ lysogens (the phage is marked with a chloramphenicol resistance cassette). Mean values from three independent experiments are presented with standard deviations shown. *P*-value is 0.67. (**C**) Images of BREX+ and BREX- cultures infected (MOI = 1) with phage λ induced from BREX+ lysogens. Scale bar, 2 μm.

Phages collected after the induction of BREX+ lysogens were used to infect non-lysogenic BREX+ and BREX- cultures. Phage induced from BREX+ culture efficiently infected and lysed BREX+ cells in liquid cultures (Figure 4A). In addition, no difference in lysogenization efficiency of BREX+ and BREX- cultures with this phage was observed (Figure 4B). Live fluorescence microscopy experiments using cells expressing SeqA-ECFP fusion also demonstrated that phage induced from BREX+ culture infected both BREX+ and BREX- cells normally (Figure 4C). We conclude that BREX+ system is unable to protect cells from phages that were generated by the BREX+ host.

### Phages that overcome BREX+ protection contain modified DNA

**Figure 5.**
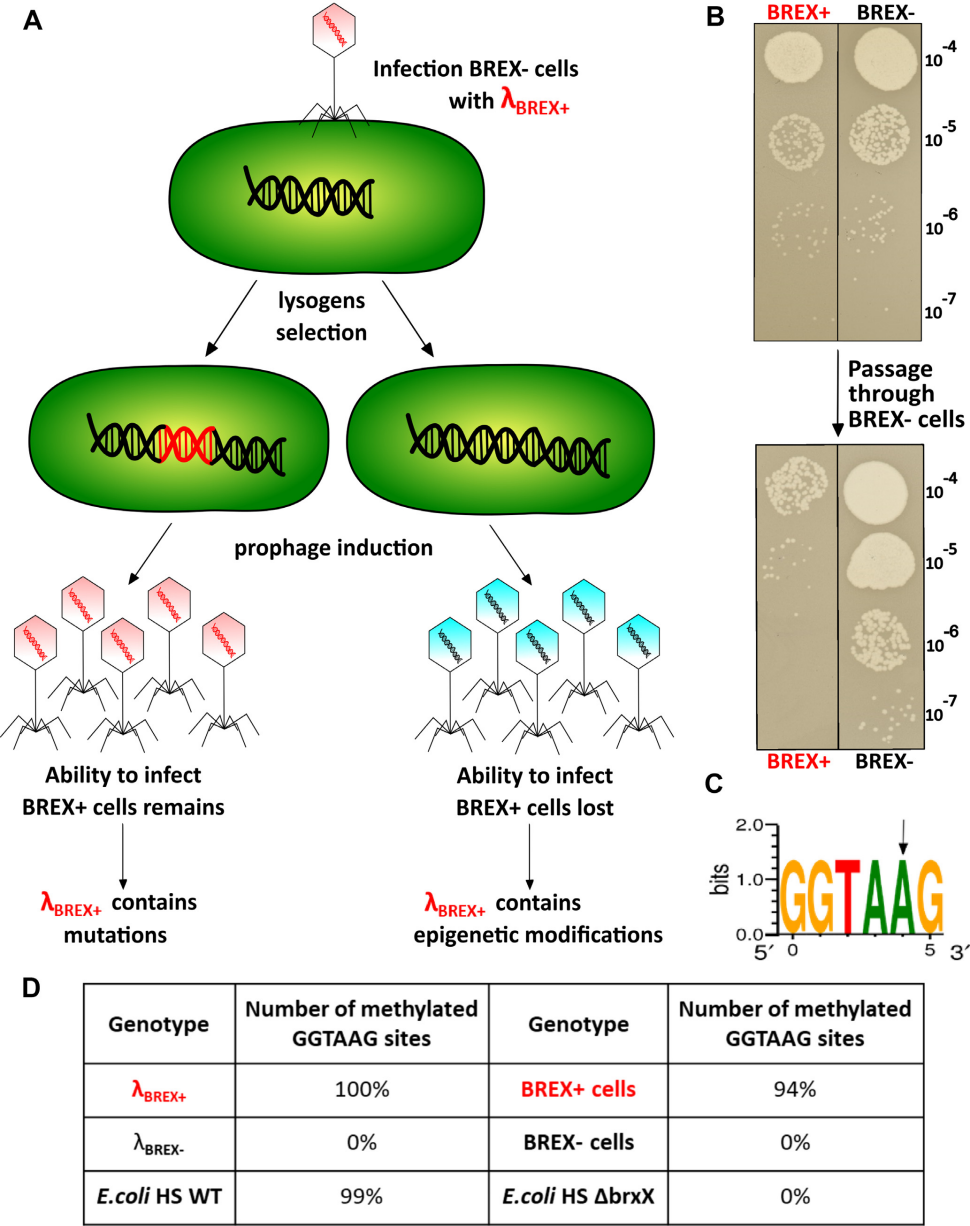
BREX system epigenetically modifies phage λ. (**A**) The scheme shows two possible ways of appearance of phages that overcome BREX action. (**B**) At the top, lawns of BREX+ and BREX- cells were spotted with indicated dilutions of λ phage lysate obtained after induction of BREX+ lysogens. Results of overnight growth at 37°C are shown. Phages from a plaque obtained on BREX+ cells were used to re-infect BREX+ or BREX- cells. The results of spotting phage progeny from these infections on BREX− and BREX+ cell lawns are shown below. (**C**) The site modified in genomic DNA of *Escherichia coli* HS cells, *E. coli* BW25113 containing the pBREXAL plasmid and in the genome of phage λ induced from BREX+ lysogens. The arrow shows the site of BREX-dependent methylation. (**D**) Statistics of BREX site modification in BREX+ cells genome and in phage induced from these cells.

Progeny phage formed after infection of BREX- cells with phages induced from BREX+ lysogens (Figure 5A) was no longer able to infect BREX+ cells efficiently (Figure 5B). In contrast, progeny phage produced after the infection of BREX+ cells continued to infect BREX+ cells well (Figure 5B). The result suggests that phages induced from BREX+ lysogens contain an epigenetic modification. DNA prepared from phage virions induced from BREX+ lysogens was sequenced on a PacBio platform that detects a range of base modifications (34). The results showed that all 18 GGTAAG sites present in the λ genome were methylated at the fifth adenosine residue (Figure 5C and D). No such modification was present in DNA of phages induced from BREX- cells. Sequencing of BREX+ cells DNA showed that 94% of the 1708 genomic GGTAAG sites were also modified. No other BREX+ specific modifications were detected. It is worth noting that adenine in the CTTACC sequence complementary to the GGTAAG site remained unmodified. Genomic DNA was also isolated from *E. coli* HS strain and the *brxX* putative methyltraferase mutant. PacBio sequencing revealed that the fifth residue of the GGTAAG motif was modified in the wild-type strain while no modification was observed in the mutant (Figure 5D). We therefore conclude that (i) BREX^Ec^ methylates GGTAAG sites, (ii) the modification requires intact *brxX* and (iii) BREX^Ec^-modified phages can productively infect BREX+ cells.

**Figure 6.**
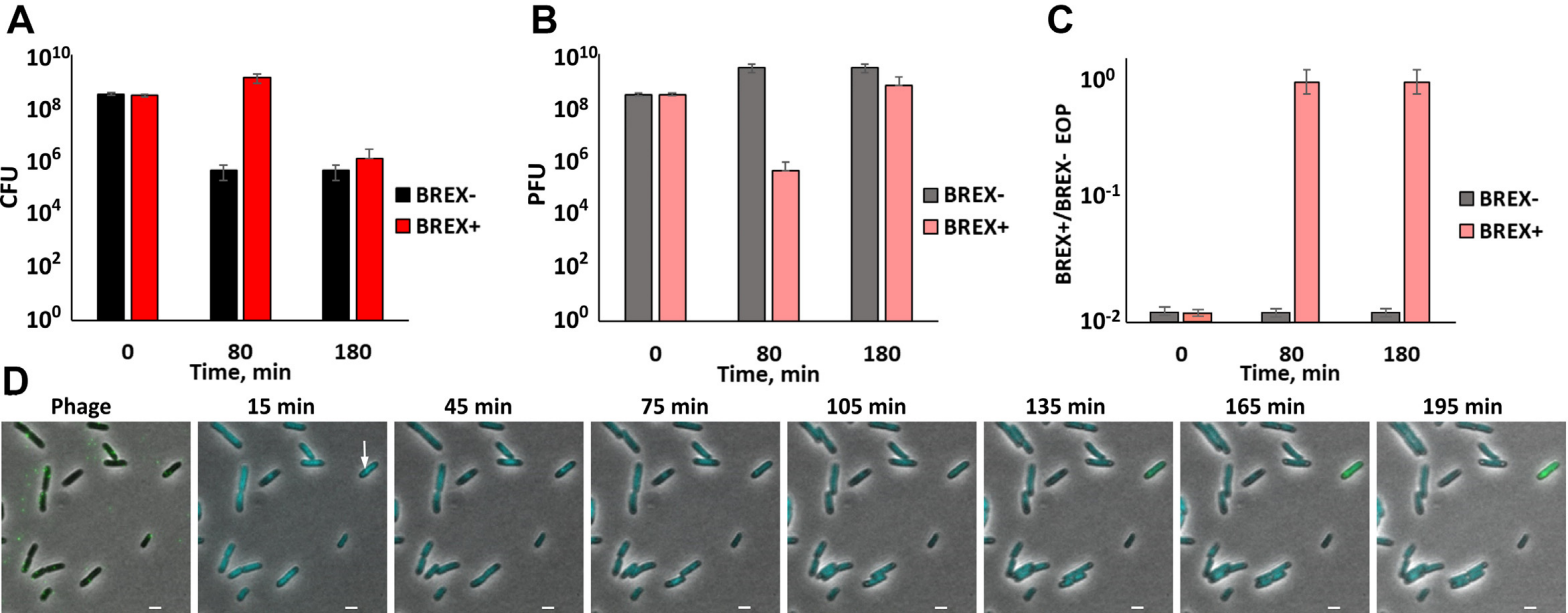
Modified phage appears in the course of infection of BREX+ cells and overcomes protection. (**A**) The amount of colony forming units (CFU) in BREX+ and BREX- cultures infected at the MOI of 1 and *t* = 0 is shown 80 and 180 min post-infection. Mean values from three independent experiments are presented with standard deviations shown. (**B**) The amount of plaque forming units (PFUs) in BREX+ and BREX- cultures from panel (A). Mean values from three independent experiments are presented with standard deviations shown. (**С**) Efficiency of plaquing (EOP) of phages collected from infected cultures from panel (B) EOP was determined by calculating the ratio of phage titer on BREX+ and BREX- cell lawns. Mean values from three independent experiments are presented with standard deviations shown. (**D**) Live microscopy observation of BREX+ cells infected (MOI = 1) with phage λ. An arrow shows a productively infected cell. Scale bar, 2 μm.

Given the epigenetic mechanism of overcoming BREX protection, we revisited infections of BREX+ cells with unmodified phage. When the number of surviving cells after infection at the MOI of 1 was monitored, there was ∼1000-times fewer survivor colonies in BREX- cultures compared to BREX+ cultures 80 min post-infection (Figure 6A). However, at later time points, the number of surviving cells became equally low in both cultures. Phage titer in infected cultures was also determined on lawns of BREX- and BREX+ cells. The ratio of these two titers, efficiency of plaquing, EOP, allows one to determine the state of phage DNA modification. Ca. 1% of initial phage used for infection was able to form plaques on BREX+ cells (see also Figure 1C). During infection of BREX- cells the overall phage titer increased but the proportion of phages able to form plaques of BREX+ lawns remained the same (Figure 6B and C). In BREX+ cells infection, the overall phage titer decreased dramatically (10 000 times) 80 min post-infection (for comparison, the burst time on BREX- cells at these conditions, when phage titer abruptly increases, is <40 min, Figure 1D). While the titer of phage in infected BREX+ cultures decreased, all phages were able to infect BREX+ cells. At later times, phage titer grew, while the ability to infect BREX+ cells with an EOP of 1 was retained. The experiment described above was conducted with λ *cI_857_ bor:Cm* phage used throughout this work and capable of lysogenic conversion. To exclude a possibility that phages that accumulate in infected BREX+ cultures appear through lysogenization followed by induction, we repeated the experiment using λ_vir_ virulent phage (31). An identical result was obtained (Supplementary Figure S3). We therefore conclude that during the infection of BREX+ culture with unmodified phage, modified phages appear in low frequency and then proceed to overtake the population. It is the appearance of these modified phages that must be responsible for eventual lysis of BREX+ cultures shown in Figure 1D. Live fluorescence microscopy analysis is consistent with this interpretation. While most BREX+ cells did not support productive infection, ECFP foci appeared in ∼1% of cells followed by subsequent accumulation of EYFP fluorescence and cell lysis, indicating productive infection (an example of such behavior can be seen in Figure 6D).

### The role of individual *brx* genes in phage protection and DNA modification

In agreement with data obtained using the *brxX* mutant of *E. coli* HS, deletion of *brxX* from the pBREXAL plasmid abolished phage protection and DNA modification (Table 1). These effects were complemented by the introduction of a compatible *brxX* expression plasmid. The role of other *brx* genes on phage protection/DNA modification was also investigated. Since deletions of *brxZ* or *brxL* genes in the context of the pBREXAL plasmid led to multiple frame-shifting deletions in other genes, we created two compatible plasmids separately expressing *brxABC* and *brxXZL* from arabinose inducible promoters. This two-plasmid system allowed, in the presence of arabinose, the same protection of cells from phage λ infection as pBREXAL. The genome of phage λ induced from lysogenic cells containing the full complement of *brx* genes on two plasmids and grown in the presence of arabinose was modified at GGTAAG sites (Table 1). As expected, deletion of the *brxX* gene in the context of the two-plasmid system led to the absence of both protection from infection and modification of phage/host DNA. Deletion of *brxA* had no effect on either protection from phage infection or on modification of phage/host DNA. Deletions of *brxB, brxC*, and *brxZ* abolished both protection and modification. Deletion of *brxL* abolished protection from infection but had a marginal effect on phage DNA modification (17 rather than 18 GGTAAG sites modified in phage genome). The result suggests that BrxL is either required for cell protection/limiting phage infection or that the presence of a single unmodified BREX site is not sufficient to allow the recognition of foreign DNA.

**Table 1.** The state of methylation of host DNA in and ability to withstand phage infection by cells carrying BREX plasmids lacking indicated genes

Genotype	BREX methylation	BREX defense
**(BREX+)ΔX**	−	**−**
**BrxX only**	**−**	**−**
**ΔA**	**+**	**+**
**ΔB**	**−**	**−**
**ΔC**	**−**	**−**
**ΔX**	**−**	**−**
**ΔZ**	**−**	**−**
**ΔL**	**+**	**−**
**BREX+ (2 plasmids)**	**+**	**+**

The effect of removal of single *brx* genes was also studied by infecting cells at high MOI with unmodified phage λ. Phage progeny was collected and titered on BREX- and BREX+ cells. The results (Supplementary Table S3) were in complete agreement with lysogen induction experiments, showing that *brxA* and *brxL* are not necessary for the appearance of modified phages that are able to overcome BREX protection.

Individual expression of *brxA, brxB, brxC* and *brxZ* had no effect on cell growth and did not lead to protection from phage infection. Expression of *brxX* mildly inhibited cell growth and had no effect on phage infection. DNA prepared from these cells was not modified. Thus, BrxX alone is not sufficient for DNA modification by the BREX system. Interestingly, expression of *brxL* was highly toxic: cell growth ceased immediately after induction and the culture density gradually declined with time.

**Figure 7.**
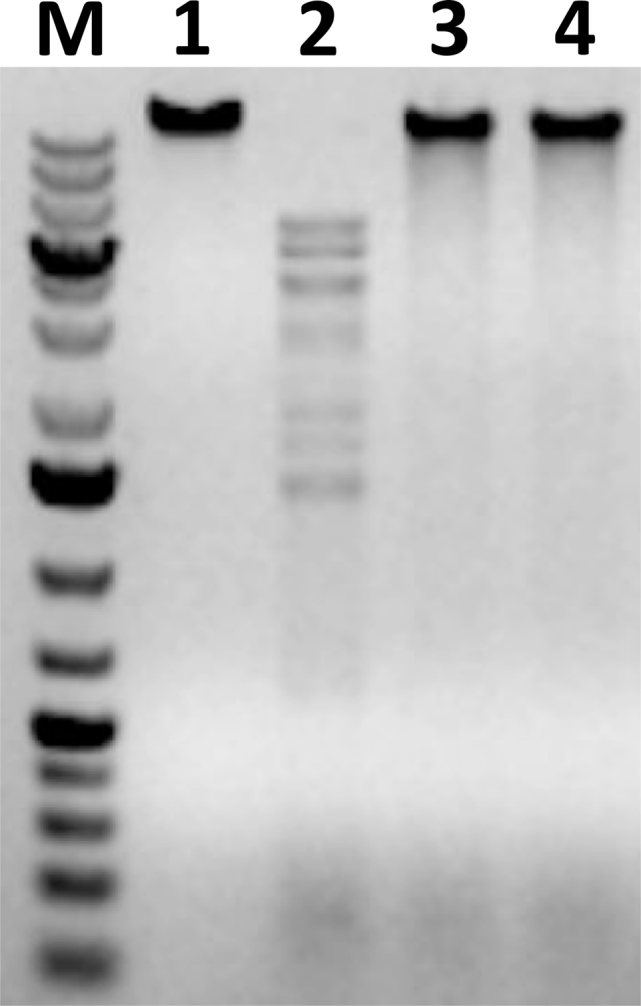
BREX+ cells extracts lack nuclease activity under conditions when restriction-endonuclease activity is detected. Cell extracts prepared from *Escherichia coli* cells harboring a plasmid containing EcoRV restriction-modification system genes (lane 2), pBREXAL plasmid (lane 3) or pBTB-2 control vector (lane 4) were combined with unmodified λ phage DNA, incubated and reaction products were resolved by agarose gel electrophoresis. Lane 1 is a control lane, phage genome incubated with buffer. **M** is a molecular weight marker.

The data presented so far are consistent with a mechanism of BREX action that is similar to the classical R-M system action, though none of the *brx* genes products have a predicted nuclease function. When extracts of BREX+ cells were combined with unmodified λ DNA, no cleavage was observed, compared to BREX- cells lysates (Figure 7). In contrast, extract of cells containing a plasmid with the EcoRV Type II R-M system (21 recognition of sites in λ genome) readily cleaved λ DNA under these conditions.

### BREX protection is circumvented by glycosylation of phage DNA

To determine the generality of the protective effect of BREX^Ec^, BREX+ cells were infected with a set of different phages: M13, Qβ, T5, T4, T7, VR5, VR7 and VpaE1 (Table 2). No protection from infection with Qβ, an RNA phage, was observed. Likewise, there was no protection against infection with M13, a single-stranded DNA phage with a double-stranded replicative intracellular form. The rest of the phages tested have double-stranded DNA genomes. The extent of BREX protection from these phages varied significantly without apparent dependence of the number of GGTAAG sites in their genomes. The apparent lack of dependence of restriction on the number of GGTAAG methylation sites is striking, considering the site number dependence in tested restriction-modification systems (35). Similar to phage λ, ∼100-fold protection from T5 and T7 infection in plaque-forming assay was observed. On the other hand, no VR7 and VpaE1 plaques were formed on BREX+ lawns, indicating very high degree of protection. In stark contrast, no protection was observed from T4 and VR5 phage infection (Table 2). The DNA of T4 is hydroxymethylated at cytosines and is additionally glycosylated (36). Restriction analysis of the VR5 phage genomic DNA confirmed that this phage also has glycosylated DNA (Supplementary Figure S4). To test if glycosylation protects from BREX system action, we assayed T4147, a mutant of T4 bearing unglycosylated hydroxymethylcytosines in its genome (23). The extent of BREX protection from T4147 phage was similar to that observed for VR7 and VpaE1 phages, which have, respectively, modified and non-modified cytosines in their unglycosylated DNA (Table 2 and Supplementary Figure S5). We conclude that phages can overcome BREX system in at least two ways: (i) GGTAAG sites methylation, which requires *brxX* and *brxBCZ*, or (ii) glycosylation of their DNA, most probably at cytosines in the opposite strand of the GGTAAG sites.

**Table 2.** Ability of BREX^Ec^ to protect cells from infections other than λ

Phage	Genome	Number of GGTAAG sites	BREX^Ec^ protection*
**Qβ**	ssRNA	3**	10^0^
**M13**	ssDNA	3***	10^0^
**T4**	dsDNA	40	10^0^
**T4147**	dsDNA	39	>10^11^
**T5**	dsDNA	65	10^2^
**T7**	dsDNA	44	10^2^
**VR5**	dsDNA	85	10^0^
**VR7**	dsDNA	66	>10^11^
**VpaE1**	dsDNA	88	>10^10^
**λ**	dsDNA	18	10^2^

*The ratio of the number of phage plaques on the BREX- lawn to the number of plaques on the BREX+ lawn.

**GGUAAG sites.

***Positive strand.

## DISCUSSION

**Figure 8.**
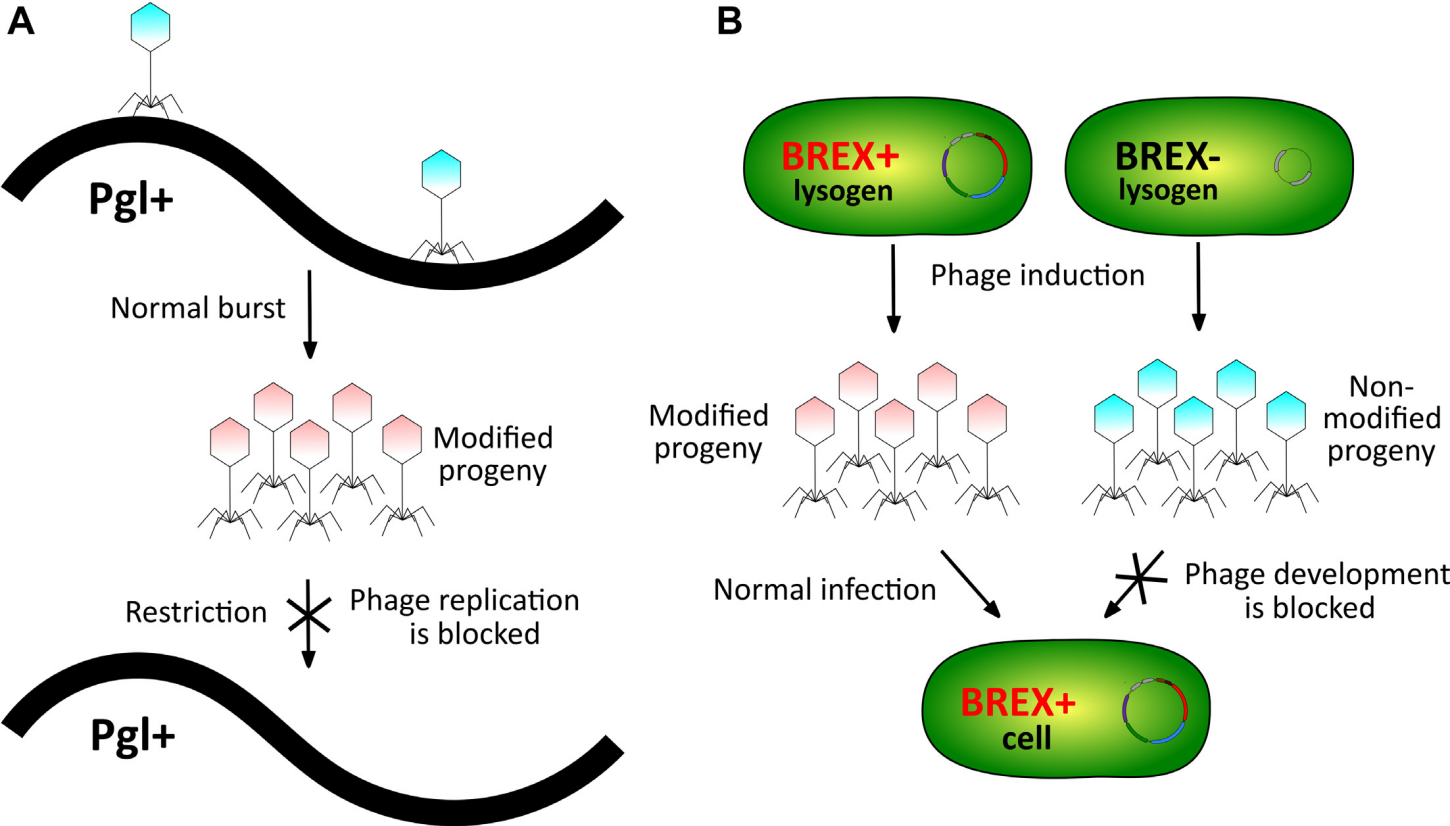
(**A**) Schematics of the Pgl mechanism based on the model by Chinenova *et al.* (13). (**B**) Schematics of the BREX^Ec^ function.

In this work, we demonstrate that *E. coli* BREX cluster protects cells from infection by diverse dsDNA phages. At least one of BREX genes, *brxA*, which codes for a protein of unknown function, is not essential for phage defense at our conditions. Of the remaining five *brx* genes, two are homologous to previously characterized Pgl system genes, encoding the putative methyltransferase BrxX (PglX) and alkaline phosphatase BrxZ (PglZ). The hallmark behavior of the Pgl system, decrease of phage yield during continuous infection but not in single-step infections, led to a proposal that the Pgl defense logic is inverted compared to that of R-M systems, where modification of phage DNA prevents restriction from happening, allowing productive infection (Figure 8A). It was further proposed the Pgl system consists of two functional modules, phage alteration/protective module *pal* and the defensive *pgl* module that blocks the development of modified phage.

Here, we show that the *pal* module of *E. coli* BREX system functions by methylating a specific asymmetric site in phage DNA. The enzymatic activity of BrxX—in the presence of BrxB, BrxC, and BrxZ—is likely responsible for this modification. Site-specific methylation of phage DNA or global glycosylation of cytosine residues allows a phage to bypass BREX defense (Figure 8B). Thus, in this regard the *E. coli* BREX system functions similarly to R-M systems and is distinct from the Pgl system. Earlier work with BREX from *B. cereus* failed to observe modification of DNA of phage progeny collected after BREX+ infection (17). The result is probably due to very low yield of phage progeny production in these experiments, which caused the authors to analyze unabsorbed/non infecting phage particles, whose genomes naturally remained unmodified. While the sites of methylation in *E. coli* and *B. cereus* BREX systems have unrelated sequences (GGTAAG and TAGGAG, respectively), there are also important commonalities—they are asymmetric, and it is an adenine in the fifth position of the recognition site that is being methylated. The site of methylation by the Pgl system, if it exists, is unknown. Phylogenetic analysis indicates that BREX systems can be divided into six types based on the presence of characteristic genes. The *E. coli* and *B. cereus* BREX systems belong to the same type I (17) and may share a common mechanism of self-versus-non-self differentiation. The Pgl system belongs to a different type, type II, which may help explain the apparent differences in reported behavior of Pgl and the two type I systems.

Our results establish the mechanism responsible for self-protection of BREX carrying cells and the means by which phages can overcome the BREX defense. Overall, the logic appears to be similar to that observed for R-M systems, where rare modified genetic invaders appear due to stochastic events, multiply and eventually take over the population of initially protected cells. It is interesting that BREX systems may undergo phase variation due to a homopolymeric tract in the *brxX* coding sequence (17) and this strategy may allow the cells to alternate the proportion of BREX+ and BREX− cells in the population which may help withstand phage predation.

For most-studied Type II R-M systems, the palindromic nature of the recognition site ensures that epigenetic protective modification is heritable. The BREX site is asymmetric, and maintenance of epigenetic marks likely require interactions between different sites, as observed for Type I and Type III systems (37–39). Our observation that phages that contain a single unmodified BREX site are able to infect BREX+ cells is consistent with this idea.

The nature of defensive action of BREX remains elusive. The λ infection must be blocked at a very early stage after the absorption of the phage. In principle, either a block of DNA injection or rapid degradation of injected DNA prior to its replication is consistent with our live microscopy data. However, rapid degradation of unmodified DNA, a mechanism that occurs in cells protected by R-M systems, is expected to decrease the transformation efficiency of plasmids carrying the protective systems genes, into naïve cells. In the absence of proper regulation, plasmids harboring known R-M systems transform naïve cells very poorly due to premature synthesis of endonuclease (40). Plasmid expressing BREX genes is transformed into naïve cells with high efficiency. Moreover, unmodified phage DNA remains stable in extracts of BREX+ cells, again arguing against rapid degradation. Identification of the defense mechanism by BREX systems will require establishment of functional interactions between essential system components, since our mutagenesis results as well as earlier data collected with the Pgl system indicate that there must be several toxin-antitoxin type interactions that allow stable maintenance of the system. Interestingly, the likely toxin in BREX, the BrxL putative protease, is absent in Pgl, suggesting that these systems may employ multiple strategies to limit viral infections.

## Supplementary Material

Supplementary Data
