# Supplementary material for: BREX system of Escherichia coli distinguishes self from non-self by methylation of a specific DNA site
Source: Nucleic Acids Res. 2018 Nov 12;47(1):253–65. doi: 10.1093/nar/gky1125 (PMC6326788; doi:10.1093/nar/gky1125)
Supplement: Supplementary Data [file gky1125_supplemental_files.docx]

**Supplementary Tables.**

**Supplementary Table S1. Primers used for cloning in this work (5’-3’ orientation).**

| **Name** | **Sequence** |
| --- | --- |
| **BREX_CM_F** | TGCATTCCAGTCTTCAGAGGGTTTTTCGATGATTAAAAATGACAAGGCATGGATGTGTAGGCTGGAGCTGCTTC |
| **BREX_CM_R** | TTAATTCACACCCAGCGCCTTATAAACAGCATCAACCGGGTCTGAGTAAAACATATGAATATCCTCCTTAG |
| **CM_check_F** | CACTCATCGCAGTACTGTTGTATTC |
| **CM_check_R** | CGCAGAATAAATAAATCCTGGTGTC |
| **BREX_check_F** | AAATTGCCTTTTCTCATGGCATCTC |
| **BREX_check_R** | GAACGTTCTTGAGCCATTGTCC |
| **Brx_pro_SacI_F** | TTATAGAGCTCCTAAAGCATGTGGTTCTTTATTTG |
| **BrxL_SphI_R** | TTATAGCATGCTTAATTCACACCCAGCGCCTTATAAAC |
| **pBTB_SphI_F** | TTATAGCATGCAACGAATTCAAGCTTGATATC |
| **pBTB_SacI_R** | TTATAGAGCTCCATACCTGACCTCCATAGC |
| **pACYC184_F** | TATATCTCGAGCCAATCAATTCTTGCGG |
| **pACYC184_R** | ATATAAGGCCTCCGGCGGTGCTTTTGCCG |
| **pBAD30_F** | TATATAGGCCTGCTAGCGGAGTGTATACTGG |
| **pBAD30_R** | ATATACTCGAGTGAGCGGATACATATTTG |
| **pTG-BREX_F** | TATATGCATGCAAGCTTGGCT |
| **pTG-BREX_R** | ATATAGAGCTCTTGGTAACGAATCAGACAATTG |
| **EcoRI_SD8_BrxA_F** | TTATATGAATTCTTTATGATTAAAAATGACAAGGCATGG |
| **BrxC_EcoRI_R** | TAAATAGAATTCTTACTGAAGGCGAATGCGATCGC |
| **BrxZ_NcoI_F** | ATAAATTCCATGGAAAAACGCGTACGCGAAGTTAC |
| **BrxL_XhoI_R** | TAA ATA CTC GAG TTAATTCACACCCAGCGCCTTATA |
| **NcoI_BrxX_F** | ATATTCCATGGATACCAATAACATTAAAAAATATG |
| **BrxZ_NcoI_R** | TAAATA ССATGG TGCTGTTATCGCTGTCAAAGCTGA |
| **delA_BglII_F** | ATATTAGATCTTCCTATCCATGCCTTGTC |
| **delA_BglII_R** | ATGATTAAAAATGACAAGGCAAGATCTAATAT |
| **delC_BglII_F** | ATATAAGATCTATTAAAGCAGGCGATCG |
| **delC_BglII_R** | TATATAGATCTTAGAGGTTTTTTAAAAATCTG |
| **delB_F1** | ATATACTGCAGGCAGTTTACCTTTTACCGG |
| **delB_R1** | GATTCAATGTTGCTGCGGGTCCCGTAGACAGGCGATATTCAAGAACGGGA |
| **delB_F2** | TCCCGTTCTTGAATATCGCCTGTCTACGGGACCCGCAGCAACATTGAATC |
| **delB_R2** | ATAGTCTGCAGCTTCAGCATCATTT |
| **delZ_F1** | ATA TAG ACG TCA CCT CTC GCG TCA AAG AG |
| **delZ_R1** | AATCATCCTGGAACGCCAGATCGATTTTAAGGCCAGCAATAAAGTCCTGA |
| **delZ_F2** | TCAGGACTTTATTGCTGGCCTTAAATCGATCTGGCGTTCCAGGATGATT |
| **delZ_R2** | GAAATG GCGCCAGCAAATGGCTG |
| **Nhe-BrxA-dir** | TATGGCTAGCATTAAAAATGACAAGGCATGG |
| **XhoStSac-BrxA*-*rev** | ATCCCTCGAGTCAGAGCTCCCGTTTTCCCTCCAGAATAG |
| **Nhe-BrxB-dir** | TATGGCTAGCATAGATCCCGTTCTTGAATATC |
| **XhoStSac-BrxB*-*rev** | ATCCCTCGAGTCAGAGCTCTTGAGGATTCAATGTTGCTG |
| **Nhe-BrxC-dir** | TATGGC AGCAATATTGAACAGATTTTTAAAAAAC |
| **XhoStSac-BrxC*-*rev** | ATCCCTCGAGTCAGAGCTCCTGAAGGCGAATGCGAT |
| **Nhe-BrxX-dir** | TATGTCTAGAAATACCAATAACATTAAAAAATATG |
| **XhoStSac-BrxX*-*rev** | ATCCGTCGACTCAGAGCTCGATCGCCTCTGGGGCATT |
| **Nhe-BrxZ-dir** | TATGGCTAGCAACTTGCAAAATCAGGACTTTA |
| **XhoStSac-BrxZ*-*rev** | ATCCCTCGAGTCAGAGCTCGAAGAAATCATCCTGGAACG |
| **Nhe-BrxL-dir** | TATGGCTAGCCAAACCCATCATGATTTACC |
| **XhoStSac-BrxL*-*rev** | ATCCCTCGAGTCAGAGCTCATTCACACCCAGCGCCTTAT |
| **TS551** | GGGAATGATATAGGGTCATTTCC |
| **TS552** | GTTGAATTAGCTGATAGCTCCTC |
| **TS578** | TAATACTAGTCGCAAGTGAATATGCAGTCAGTTTTAGAGCTAGAAATAGCAAGTTAAAATAAG |
| **TS579** | TGAACTCGAGTAGGGATAACAGG |
| **TS580** | TTTTGTCGACGGCGGAACAGCAAGAGACAAAAGCGC |
| **TS581** | AACGCGCCGTCTGGTAGAAAATCCTGCAAAGGGCAGCAGAGTGC |
| **TS582** | CCCTTTGCAGGATTTTCTACCAGACGGCGCGTTCCCCTGC |
| **TS583** | CCCTTTGCAGGATTTTCTACCAGACGGCGCGTTCCCCTGC |

**Supplementary Table S2. Primers used for sequencing (5’-3’ orientation).**

| **Brx_F_1** | GCTTGCTGGTCCGTTAAATC |
| --- | --- |
| **Brx_F_2** | AATGGGTGGAACAACTCGAC |
| **Brx_F_3** | CGATTTCTCTGCGATCAACA |
| **Brx_F_4** | CGCTGGTTGAACATATTCGT |
| **Brx_F_5** | AAAAATATGCCCCACAGGC |
| **Brx_F_6** | GGTTACCGTACCCGTGACAT |
| **Brx_F_7** | CTCTTGGAACTCGTGCTGTG |
| **Brx_F_8** | ACGAGTGGTTTGAGGACGAC |
| **Brx_F_9** | CGCCTCATGAACAAGACTGG |
| **Brx_F_10** | GATGATCCTCAAGAGCCTGG |
| **Brx_F_11** | GCAGAAAAACAGCCGCAG |
| **Brx_F_12** | CGTGATCAGTGGATCGATGT |
| **Brx_F_13** | GACTGCCGGTAATGGTAAGC |
| **Brx_R_1** | GTACTAATGGCCAGGCGTTG |
| **Brx_R_2** | ACTGACCGACTTCATCCACC |
| **Brx_R_3** | AGTCTTGCTTGTTAGCCGGA |
| **Brx_R_4** | GTTTTTCCCCGGATTGTTTC |
| **Brx_R_5** | TTATCTGCAGCGATCCCAAG |
| **Brx_R_6** | AGCATCAGCATCGCAAATC |
| **Brx_R_7** | AATTTATGCTGACTTCCGGC |
| **Brx_R_8** | CTTTCGGCGATAAATTCGAG |
| **Brx_R_9** | CAGCGTGTTCAGGGTGATG |
| **Brx_R_10** | CTTCGTTCCAGTTACGGCTT |
| **Brx_R_11** | CGTCTGCAACAGGCTCACT |
| **Brx_R_12** | CTCCACGAACGGGATCATAC |
| **Brx_R_13** | GCTTACCCGATTCAAATTGC |
| **pBTB_F** | GCACGGCGTCACACTTTGC |
| **pBTB_R** | CGAAGCGAGCCAGCCGG |
| **pBAD_F** | ATGCCATAGCATTTTTATCC |
| **pBAD_R** | GATTTAATCTGTATCAGG |

**Supplementary Table S3. Bacterial strains and plasmids used in this work.**

| ***E. coli* strain** | **Comments** | **Source** |
| --- | --- | --- |
| HS | Natural isolate with BREX^Ec^, *Str^R^* | Paul Cohen |
| HS ∆BREX^Ec^ | Natural isolate with deleted BREX^Ec^, *Str^R^, Cm^R^* | This work |
| BW26113 | Strain for routine work | Lab stock |
| JM110 | Dam- stain for PacBio sequencing | Lab stock |
| MC4100 lysogen | Source of λ *cI*_857_ *bor::Cm^R^, Cm^R^* | Ryland Young |
| MG1655 seqA-ECFP Dam- | SeqA-experiment | Lanying Zeng |
| LE392 | *sup^E^* and *sup^F^* host | Lanying Zeng |
| LE392(λ_LZ1_) | Prophage induction experiment, *Kan^R^* | Lanying Zeng |
| MG1655 (λD-EYFP *cI_857_* *bor::Kan^R^*) [pBR322-PLate*D] | Source of λD-EYFP *cI*_857_ *bor::Kan^R^, Kan^R^* | Lanying Zeng |
| BW26113 [pEF42] | Strain with the EcoRV system, *Amp^R^* | Lab stock |
| **Plasmids** |  |  |
| pBREXAL | Plasmid with the BREX^Ec^ system under a natural promoter, *Kan^R^* | This work |
| pTG-BREX | Plasmid with the BREX^Ec^ system under a natural promoter, *Cm^R^* | This work |
| pTG | Plasmid with the h15A origin, arabinose promoter and *Cm^R^* | This work |
| pBREX1 | Arabinose-inducible plasmid with *brxABC, Kan^R^* | This work |
| pBREX2 | Arabinose-inducible plasmid with *brxXZL, Amp^R^* | This work |
| pBREX1∆N, N=A,B,C | Arabinose-inducible pBREX1 plasmid with deletion of N gene | This work |
| pBREX2∆N, N=X,Z,L | Arabinose-inducible pBREX2 plasmid with deletion of N gene | This work |
| pBAD-brxN, N=A,B,C,X,Z,L | Arabinose-inducible plasmid expressing an individual *brx* gene, *Amp^R^* | This work |

**Supplementary Table S4.**

| **Genotype** | **BREX methylation detected by PacBio in induced phage genomes and bacterial DNA** | **Modified progeny after incubation with unmodified phage** |
| --- | --- | --- |
| **∆A** | **+** | **+** |
| **∆B** | **-** | **-** |
| **∆C** | **-** | **-** |
| **∆X** | **-** | **-** |
| **∆Z** | **-** | **-** |
| **∆L** | **+** | **-** |
| **BREX+**  **(2 plasmids)** | **+** | **+** |


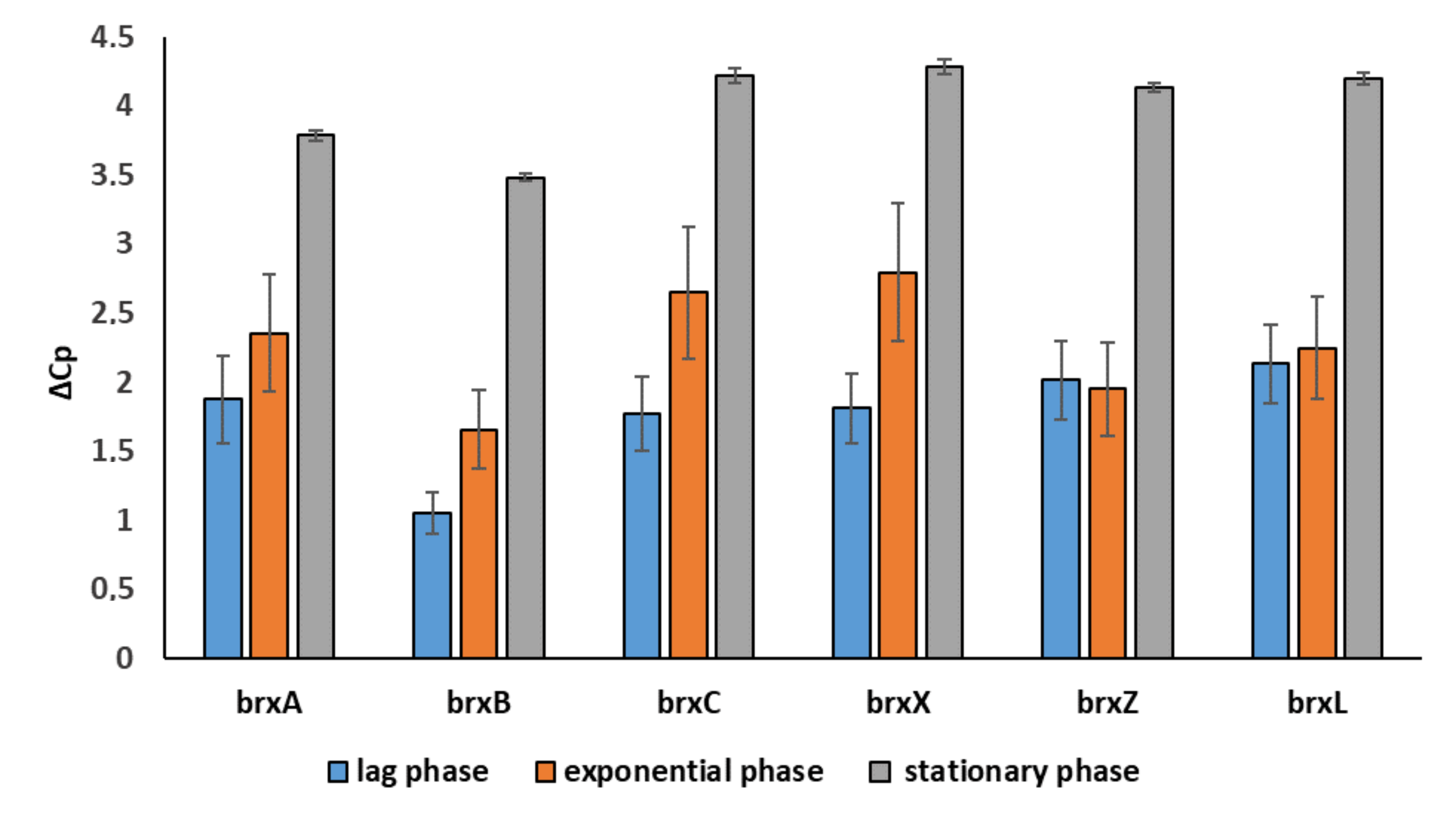


**Supplementary Figure S1. Expression of the BREX^Ec^ cluster genes in *Escherichia coli* HS during laboratory growth.**

Aliquots of *E. coli* HS culture grown in LB medium at 37 ^o^C were taken at lag, exponential, and stationary phases of growth (OD_600_ = 0.15, 0.6, and 3.0, respectively); total RNA was extracted and qPCR reactions were performed with primers specific for indicated *brx* genes using cDNA prepared with random primers. ∆Cp values were calculated as a difference between Cp of essential *rho* gene and each *brx* gene. Since the difference was less than 0, for convenience, 5 was added to all ∆Cp values. Mean values and standard deviations obtained from three experiments are presented.


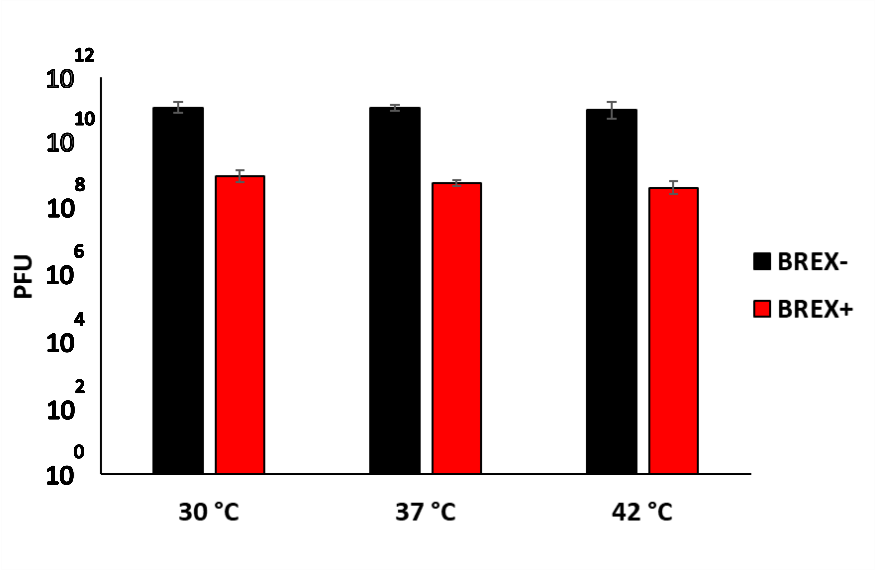


**Supplementary Figure S2. Efficiency of BREX^Ec^ protection at different temperatures.**

A virulent strain of phage λ_vir_ was titered on BREX- and BREX+ lawns and plaques were counted after overnight incubation at 30 °C, 37 °C, and 42 °C.


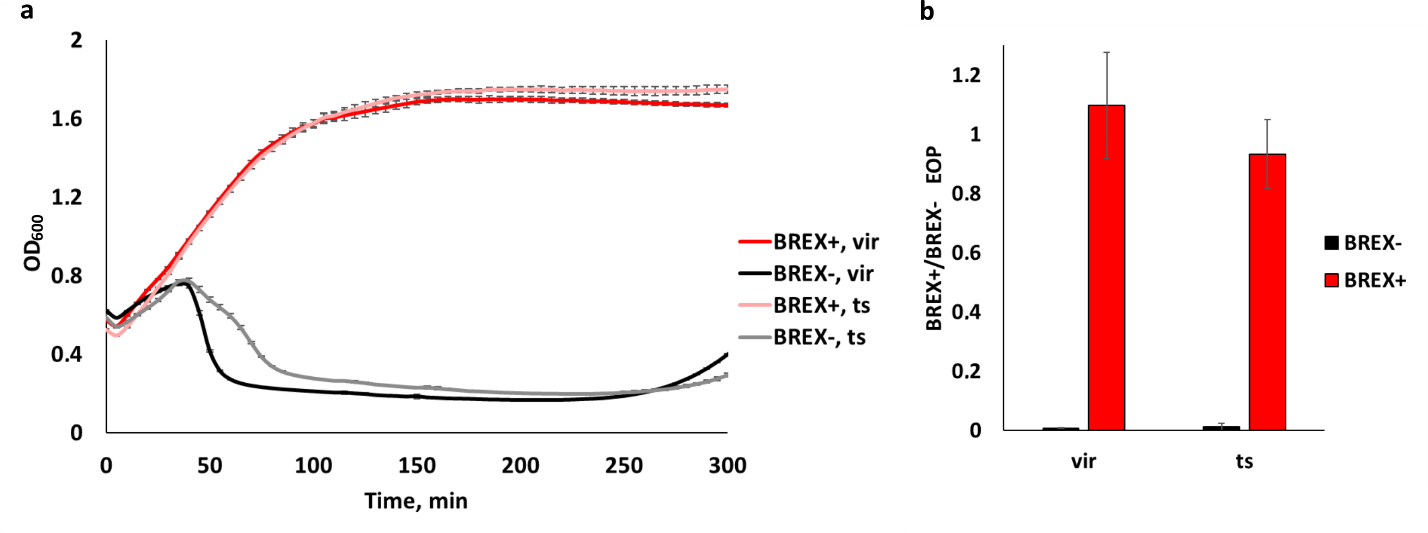


**Supplementary Figure S3. Efficiency of BREX^Ec^ protection against λ_vir_ and temperature against λ_ts_ phages (the latter capable of lysogenisation).**

**a.** Growth curves of BREX+ and BREX- cultures during infection with two strains of λ phage at MOI = 1. Phage was added at t=0. Each growth curve shows mean optical density values and standard deviations obtained from three independent experiments.

**b**. Efficiency of plaquing (EOP) of phages collected from infected cultures from panel **a** after overnight incubation. EOP was determined by calculating the ratio of phage titer on BREX+ and BREX- cell lawns. Mean values from three independent experiments are presented with standard deviations shown.


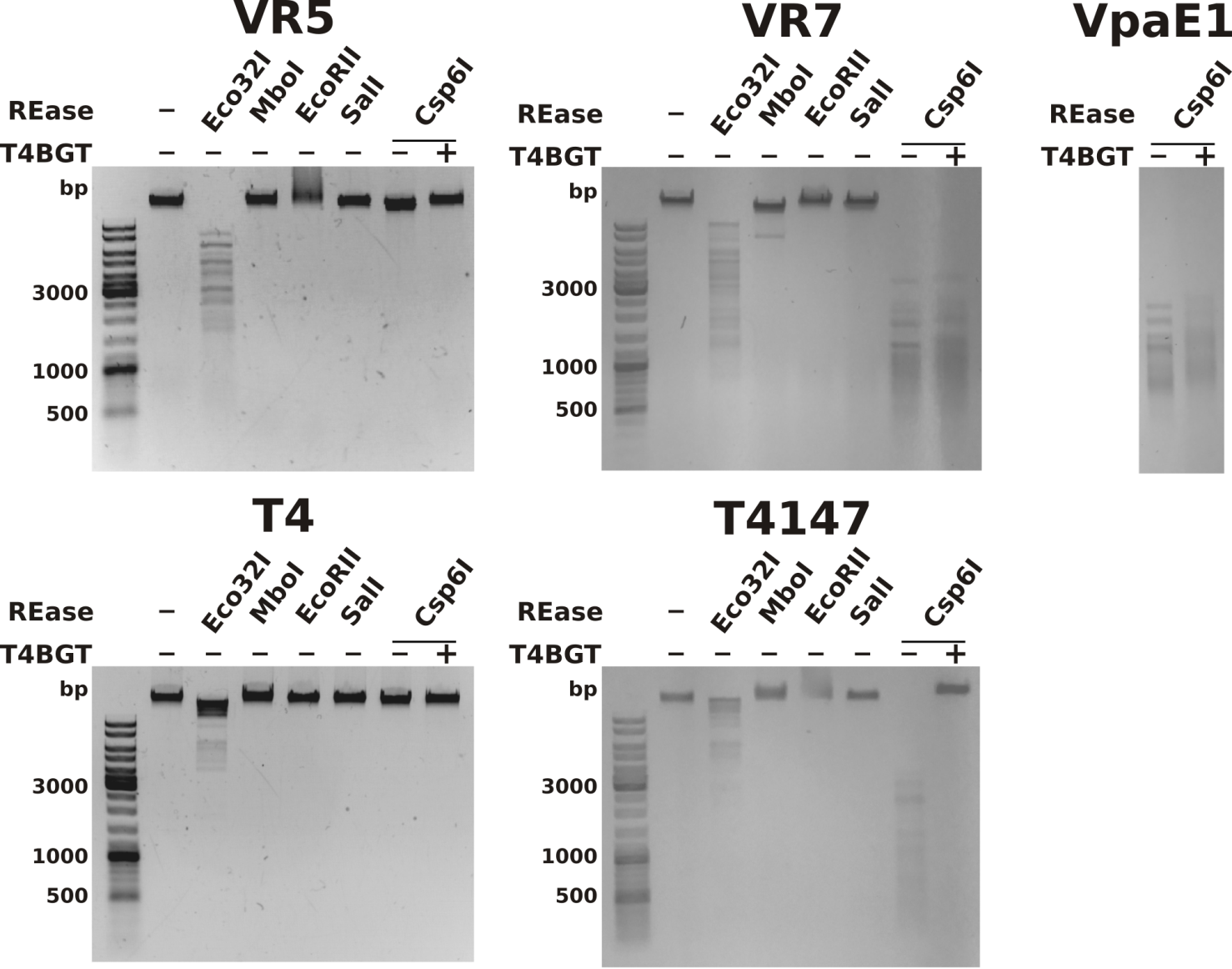


**Supplementary Figure S4. Modification state of phage DNA**. Genomic DNA of VR5 (1, 2), VR7(1–3), VpaE1(4), T4, and T4147(5) phages were digested with restriction endonucleases (REase). The genomic DNA was either incubated (**+**) or not incubated (-) with T4 beta-glucosyltransferase (T4BGT) prior restriction nuclease treatment. Eco32I is insensitive to modifications; MboI - cleavage is blocked or impaired by Dam methylation; EcoRII - cleavage is blocked or impaired by Dcm methylation; SalI - cleavage is blocked or impaired by CpG methylation; Csp6I – insensitive to methylation (and hydroxymethylation) but sensitive to glycosylation. Restriction endonuclease analysis shows that DNA of VR5 and T4 is glycosylated, T4174 is hydoxymethylated, and VR7 has modified cytosines. Detailed restriction endonuclease analysis of VpaE1 could be found (4) where is shown that DNA of VpaE1 is not modified.

**
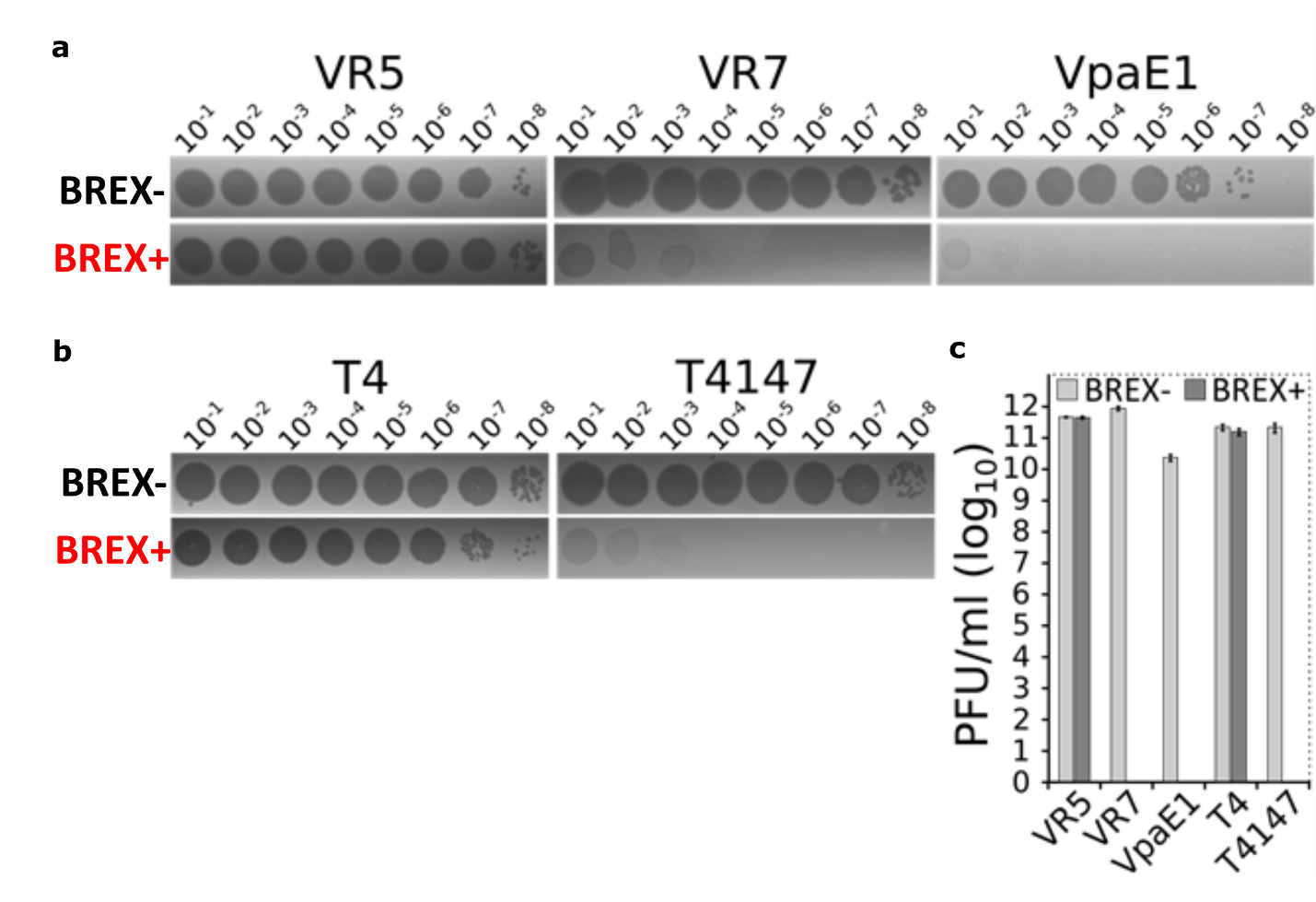
**

**Supplementary Figure S5. Phages with glycosylated or BREX-modified DNA overcome BREX protection.**

**a.** Only glycosylated VR5 phage escapes BREX protection, while infection of modified VR7 and non-modified VpaE1 phage is arrested by BREX.

**b.** Glycosylated T4 phage escapes BREX protection, while BREX fully protects against infection of T4147 phage that is a mutant of T4 lacking glycosyl moiety on its hydroxymethylcytosines.

**c.** Efficiency of plaquing of VR5, VR7, VpaE1, T4, and T4147 phages was determined on BREX- and BREX+ bacterial lawns. VR7, VpaE1, and T4147 did not form plaques on BREX+ lawn; therefore, number of PFUs was not determined. Lysis zones of these phages on BREX+ lawn visible in **a, b** is probably due to the nonproductive lysis from without.

**Supplementary references**

1. Kaliniene,L., Klausa,V. and Truncaite,L. (2010) Low-temperature T4-like coliphages vB_EcoM-VR5, vB_EcoM-VR7 and vB_EcoM-VR20. *Arch. Virol.*, **155**, 871–880.

2. Kaliniene,L., Zajančkauskaitė,A., Šimoliūnas,E., Truncaitė,L. and Meškys,R. (2015) Low-temperature bacterial viruses VR - a small but diverse group of E. coli phages. *Arch. Virol.*, **160**, 1367–1370.

3. Kaliniene,L., Klausa,V., Zajančkauskaite,A., Nivinskas,R. and Truncaite,L. (2011) Genome of low-temperature T4-related bacteriophage vB_EcoM-VR7. *Arch. Virol.*, **156**, 1913–1916.

4. Šimoliūnas,E., Vilkaitytė,M., Kaliniene,L., Zajančkauskaitė,A., Kaupinis,A., Staniulis,J., Valius,M., Meškys,R. and Truncaitė,L. (2015) Incomplete LPS core-specific felix01-like virus vB_EcoM_VpaE1. *Viruses*, **7**, 6163–6181.

5. Bair,C.L. and Black,L.W. (2007) A Type IV Modification Dependent Restriction Nuclease that Targets Glucosylated Hydroxymethyl Cytosine Modified DNAs. *J. Mol. Biol.*, **366**, 768–778.
